# New clinical data on human spinal cord re-irradiation tolerance

**DOI:** 10.1007/s00066-021-01772-7

**Published:** 2021-05-05

**Authors:** Hiroshi Doi, Keisuke Tamari, Ryoong-Jin Oh, Carsten Nieder

**Affiliations:** 1 Miyakojima IGRT Clinic, 1-16-22 Miyakojimahondori, 534-0021 Osaka, Miyakojima-ku Japan; 2 Department of Radiation Oncology, Kindai University Faculty of Medicine, 377-2 Ohno-Higashi, 589-8511 Osaka, Osaka-Sayama Japan; 3 Department of Radiation Oncology, Osaka University Graduate School of Medicine, 2-2 Yamadaoka, 565-0871 Osaka, Suita Japan; 4 Department of Oncology and Palliative Medicine, Nordland Hospital Trust, 8092 Bodø, Norway; 5 Department of Clinical Medicine, Faculty of Health Sciences, UiT—The Arctic University of Norway, Tromsø, Norway

**Keywords:** Spinal metastases, Palliative radiotherapy, Radiation myelopathy, Dose constraint, Treatment planning

## Abstract

**Purpose:**

To provide additional clinical data about the re-irradiation tolerance of the spinal cord.

**Methods:**

This was a retrospective bi-institutional study of patients re-irradiated to the cervical or thoracic spinal cord with minimum follow-up of 6 months. The maximum dose (Dmax) and dose to 0.1cc (D0.1cc) were determined (magnetic resonance imaging [MRI]-defined cord) and expressed as equivalent dose in 2‑Gy fractions (EQD2) with an α/β value of 2 Gy.

**Results:**

All 32 patients remained free from radiation myelopathy after a median follow-up of 12 months. Re-irradiation was performed after 6–97 months (median 15). In 22 cases (69%) the re-irradiation spinal cord EQD2 Dmax was higher than that of the first treatment course. Forty-eight of 64 treatment courses employed fraction sizes of 2.5 to 4 Gy to the target volume. The median cumulative spinal cord EQD2 Dmax was 80.7 Gy, minimum 61.12 Gy, maximum 114.79 Gy. The median cumulative spinal cord D0.1cc EQD2 was 76.1 Gy, minimum 61.12 Gy, maximum 95.62 Gy. Besides cumulative dose, other risk factors for myelopathy were present (single-course Dmax EQD2 ≥51 Gy in 9 patients, single-course D0.1cc EQD2 ≥51 Gy in 5 patients).

**Conclusion:**

Even patients treated to higher cumulative doses than previously recommended, or at a considerable risk of myelopathy according to a published risk score, remained free from this complication, although one must acknowledge the potential for manifestation of damage in patients currently alive, i.e., still at risk. Individualized decisions to re-irradiate after appropriate informed consent are an acceptable strategy, including scenarios where low re-irradiation doses to the spinal cord would compromise target coverage and tumor control probability to an unacceptable degree.

## Introduction

Experimental animal data have suggested that spinal cord re-irradiation is a feasible approach [1, 2]. In clinical practice, several treatment planning and delivery techniques allow for sparing of the spinal cord [3–8]. However, it is not always possible to avoid limited re-irradiation doses, and sometimes high-dose re-irradiation is the only treatment option [9]. A simple method for calculating re-irradiation tolerance is to assume time-dependent recovery (25% after 6 months, 50% after 12 months), resulting in tolerance doses of 125% and 150%, respectively [10]. For example, an initial treatment course that resulted in an equivalent spinal cord dose (EQD2) close to 50 Gy in 2‑Gy fractions may be supplemented by a second course with spinal cord EQD2 of 25 Gy in 2‑Gy fractions 18 months later, i.e., 50% of the tolerance dose in the first-line setting. For occasional patients, such limited re-irradiation doses are not sufficient to achieve the desired outcome in terms of efficacy or local control [11]. A myelopathy risk score has been developed to inform treatment planning decisions under these special circumstances. The development cohort included 40 individual patients from eight different publications, and 11 of these patients had developed radiation myelopathy [12]. Later, 38 additional patients treated by the authors of that report or published in four different other publications were studied [13]. The risk score based on three variables (cumulative equivalent dose, highest equivalent dose of all treatment series in a particular individual, and time interval between first and second course), which discriminates three different risk groups, did not require modification after evaluation of these 38 new patients. Still <5% of the patients in the low-risk group had developed radiation myelopathy. A recent publication from Japan (74 patients) has provided additional clinical data [14]. In the present bi-institutional study, all Japanese data fulfilling certain eligibility criteria were pooled with new data collected by the first author of the risk score in order to confirm the safety of the previous recommendations. Neither institution has encountered any case of re-irradiation myelopathy in the cervical or thoracic spine so far.

## Materials and methods

For the present retrospective study, the Japanese authors provided expanded individual patient data that originated from their previous publication [14]. The Norwegian data, which have not been published previously, were extracted and converted to EQD2 according to the same methods. The Institutional Review Board at Miyakojima IGRT clinic approved the study. Only patients who were followed for at least 6 months from re-irradiation to the cervical or thoracic spinal cord were eligible. The follow-up information, e.g., clinical symptoms of radiation myelopathy, was obtained from the institutional electronic patient records. Re-irradiation was performed in the time period between 2007 and 2018, with different treatment planning systems and linear accelerators in use. Image guidance, e.g., cone beam computed tomography (CBCT), was not mandatory. Fractionation and total dose were at the discretion of the treating team of radiation oncologists and physicists. Spinal cord EQD2 was calculated according to the linear-quadratic model with an α/β value of 2 Gy [15, 16], based on the dose–volume histograms of the three-dimensional treatment plans where the true spinal cord had been contoured, as opposed to surrogate structures such as the spinal canal. Both maximum dose (Dmax) and dose to 0.1 cc (D0.1cc) of the re-irradiated spinal cord were tabulated. In case of two-dimensional treatment techniques, spinal cord doses were reconstructed on fused computed tomography (CT) and magnetic resonance imaging (MRI) scans. Coregistered treatment planning scans from the first and second courses were used to assess the cumulative doses in the overlapping region. The risk score was calculated as described previously [12, 13] (displayed in Table 1).

**Table 1:** Risk factors for radiation myelopathy after re-irradiation

Risk factor	Characteristic	Points
Time interval	<6 months	4.5
	≥6 months	0
EQD2 for first or second course	≥51 Gy	4.5
EQD2 for both courses	<51 Gy	0
Cumulative EQD2, both courses	60.1–65 Gy	1
	65.1–70 Gy	2
	70.1–75 Gy	3
	75.1–80 Gy	4
	80.1–85 Gy	5
	85.1–90 Gy	6

*EQD2* equivalent dose in 2‑Gy fractions. For doses >90 Gy the same principle of adding one point per dose interval applies. Low risk: point sum 0‑3, intermediate risk: point sum 4–6, high risk: point sum >6.

## Results

Sixteen adult patients received re-irradiation at the Norwegian center and 16 at the Japanese center. All 32 patients were re-irradiated to the cervical or thoracic spinal cord and were followed for at least 6 months from re-irradiation (median 12 months, maximum 90 months), Table 2. The median age was 60.5 years. Twenty-four patients (75%) were re-irradiated for bone metastases (diagnosis code C79.5). Other indications included multiple myeloma, leptomeningeal spinal metastases, and intrathoracic primary tumors, among others. The most common primary tumors were lung cancer (C34.9) in 10 patients (31%), hepatic cancer (19%), and kidney cancer (12.5%). Re-irradiation was performed after 6–97 months (median 15). The cervical cord was re-irradiated in 9 patients (28%), including those with treatment of the cervicothoracic region such as level C7-Th2. A single vertebra was re-irradiated in 12 patients (37.5%), two in 9 patients (28%), and three or more in 11 patients (34%).

**Table 2:** Patient characteristics (1–16 Norway, 17–32 Japan)

Number	Gender Follow-up (months)	Age^d^ (years)	Primary tumor Interval to reRT (months)	Secondary diagnosis	Treated region	Dose per fraction (Gy), number of fractions	Technique	Spinal cord Dmax	Spinal cord D0.1cc	EQD2 Dmax (Gy)	EQD2 D0.1cc (Gy)	Cumulative EQD2 Dmax (Gy)	Cumulative EQD2 D0.1cc (Gy)	Risk score (points) Risk group
1	Female	53	C54.2	C79.5	Th11/12	3 × 13	3‑D conformal	101%	101%	49.53	49.53	81.26	81.26	5
	9	–	16	–	Th11/12	3 × 10	2‑D dose reconstructed	90%	90%	31.73	31.73	–	–	Intermediate
2	Male	65	C18.9	C79.5	Th12-L2	3.5 × 10	2‑D dose reconstructed	87%	87%	38.41	38.41	68.49	68.49	2
	11	–	14	–	Th12-L2	3 × 10	2‑D dose reconstructed	87%	87%	30.08	30.08	–	–	Low
3	Male	54	C64.9	C79.5	Th4‑6	3 × 10	2‑D dose reconstructed	89%	89%	31.17	31.17	62.34	62.34	1
	48	–	18	–	Th4‑6	3 × 10	2‑D dose reconstructed	89%	89%	31.17	31.17	–	–	Low
4	Female	67	C25.9	C77.2	Paraaortic nodes (Th 12)	2.5 × 15	2‑D dose reconstructed	98%	98%	40.88	40.88	73.16	73.16	3
	6	–	10	–	Paravertebral (Th12)	3 × 10	3‑D conformal	91%	91%	32.28	32.28	–	–	Low
5	Female	53	C34.9	–	Mediastinum (Th12)	2.8 × 15	3‑D conformal	93%	93%	44.96	44.96	78.96	78.96	4
	6	–	6	C79.3	Th12-S2	4 × 7	2‑D dose reconstructed	88%	88%	34.00	34.00	–	–	Intermediate
6	Female	64	C34.9	–	Right lung (Th5-8)	8.5 × 2	2‑D dose reconstructed	95%	95%	40.68	40.68	61.12	61.12	1
	11	–	9	–	Right lung (Th5-8)	2.8 × 15	3‑D conformal	55%	55%	20.44	20.44	–	–	Low
7	Male	55	C34.9	C79.5	C5‑7	2 × 15 + SIB 0.7 × 15	3‑D conformal	97%^a^	97%	28.66	28.66	61.50	61.50	1
	24	–	8	–	C5‑7	3 × 10	2‑D dose reconstructed	92%	92%	32.84	32.84	–	–	Low
8	Female	61	C04.9	C79.5	C2‑5	8 × 1	2‑D dose reconstructed	99%	99%	19.64	19.64	64.95	64.95	1
	9	–	26	–	Head & neck (C2-3)	2 × 35	3‑D conformal	99%^b^	99%	45.31	45.31	–	–	Low
9	Male	50	C15.5	C77.1	Mediastinum	3 × 10	3‑D conformal	85%	85%	29.01	29.01	63.72	63.72	1
	14	–	32	–	Esophagus (Th6-7)	2 × 25	3‑D conformal	78%	78%	34.71	34.71	–	–	Low
10	Male	81	C34.9	C79.5	Th5‑8	2.8 × 7	2‑D dose reconstructed	92%	92%	20.63	20.63	72.64	72.64	7.5
	50	–	20	–	Mediastinum	2.8 × 15	3‑D conformal	102%	102%	52.01	52.01	–	–	High
11	Female	54	C50.9	C79.5	Th11-L1	4 × 5	2‑D dose reconstructed	101%	101%	30.50	30.50	67.40	67.40	2
	10	–	13	–	Th11-L1	3 × 10	2‑D dose reconstructed	99%	99%	36.90	36.90	–	–	Low
12	Female	68	C90.0	–	Th6-10	2.8 × 10	2‑D dose reconstructed	96%	96%	31.50	31.50	67.81	67.81	2
	8	–	12	–	Th8-10	3 × 10	2‑D dose reconstructed	98%	98%	36.31	36.31	–	–	Low
13	Male	58	C34.9	C77.1	Mediastinum (Th7-8)	2 × 15 + boost 2.5 × 3	3‑D conformal	97%^c^	97%	33.15	33.15	68.77	68.77	2
	7	–	8	–	Mediastinum	2.8 × 15	3‑D conformal	80%	80%	35.62	35.62	–	–	Low
14	Female	55	C50.9	C79.5	C6-Th4	3 × 10	2‑D dose reconstructed	98%	98%	36.31	36.31	74.41	74.41	3
	57	–	27	–	C7-Th5	3 × 10	2‑D dose reconstructed	101%	101%	38.10	38.10	–	–	Low
15	Male	82	C90.0	–	C7-Th2	3 × 10	3‑D conformal	101%	101%	38.10	38.10	73.82	73.82	3
	16	–	50	–	C6-Th3	3 × 10	2‑D dose reconstructed	97%	97%	35.72	35.72	–	–	Low
16	Male	73	C34.9	–	Mediastinum	3 × 9	3‑D conformal	98%	98%	32.68	32.68	82.76	82.76	5
	6	–	13	–	Mediastinum	1.5 × 2 (BID) x15 + boost 2 × 4	3‑D conformal	Different	Different	50.08	50.08	–	–	Intermediate
17	Male	60	C64.9	C79.5	C1‑3	2 × 20	3‑D conformal	99%	98%	39.40	38.81	108.86	89.21	14.5 for Dmax, 6 for D0.1cc
	61	–	29	–	C2‑3	4.5 × 10	IMRT	97%	80%	69.46	50.40	–	–	High (Dmax), intermediate
18	Male	60	C73.9	C79.5	Th2‑6	3 × 12	Single beam	112%	112%	54.03	54.03	104.43	95.53	13.5 for Dmax, 12.5 for D0.1cc
	18	–	97	–	Th3‑5	4.5 × 10	IMRT	80%	71%	50.40	41.50	–	–	High
19	Female	65	C34.9	C79.5	Th8	2.5 × 16	3‑D conformal	105%	105%	48.56	48.56	80.15	74.21	5 for Dmax, 3 for D0.1cc
	15	–	13	–	Th8	3 × 15	IMRT	69%	60%	31.59	25.65	–	–	Intermediate (Dmax), low
20	Male	82	C34.9	C79.5	Th7‑9	3 × 10	3‑D conformal	110%	109%	43.73	43.08	91.86	82.61	7 for Dmax, 5 for D0.1cc
	35	–	14	–	Th6‑7	5 × 10	IMRT	70%	62%	48.13	39.53	–	–	High (Dmax), intermediate
21	Female	47	C22.0	C79.5	Th7‑9	3 × 10	3‑D conformal	103%	102%	39.32	38.71	92.31	81.41	11.5 for Dmax, 5 for D0.1cc
	90	–	18	–	Th8‑9	4 × 13	IMRT	79%	69%	52.99	42.70	–	–	High (Dmax), intermediate
22	Male	74	C22.0	C79.5	Skull base-C4	3 × 10	3‑D conformal	99%	99%	36.90	36.90	87.68	80.88	6 for Dmax, 5 for D0.1cc
	7	–	7	–	C2	3 × 20	IMRT	78%	71%	50.78	43.98	–	–	Intermediate
23	Male	67	C22.0	C79.5	Th12	2 × 20	3‑D conformal	61%	55%	19.64	17.05	77.17	72.15	8.5 for Dmax, 7.5 for D0.1cc
	8	–	12	–	Th12	5 × 10	IMRT	78%	76%	57.53	55.10	–	–	High
24	Male	82	C22.0	C79.5	C7-Th2	3 × 10	Single beam	103%	103%	39.32	39.32	82.37	77.83	5 for Dmax, 4 for D0.1cc
	12	–	20	–	Th1‑3	3 × 20	IMRT	70%	65%	43.05	38.51	–	–	Intermediate
25	Male	64	C34.9	C79.5	Th9-10	3 × 10	Single beam	105%	105%	40.56	40.56	114.79	95.62	15.5 for Dmax, 12.5 for D0.1cc
	8	–	70	–	Th8-10	4 × 15	IMRT	89%	74%	74.23	55.06	–	–	High
26	Male	43	C34.9	–	Mediastinum	2 × 30	3‑D conformal	68%	67%	34.27	33.57	97.65	86.29	12.5 for Dmax, 10.5 for D0.1cc
	20	–	38	C79.5	Th2	4.2 × 13	IMRT	84%	75%	63.38	52.72	–	–	High
27	Male	72	C22.0	C79.5	Th2‑3	2 × 23	3‑D conformal	105%	104%	49.51	48.80	95.39	88.20	8 for Dmax, 6 for D0.1cc
	12	–	35	–	Th2	3 × 20	IMRT	73%	66%	45.88	39.40	–	–	High (Dmax), intermediate
28	Male	57	C64.9	C79.5	C2-Th2	3 × 10	3‑D conformal	102%	102%	38.71	38.71	87.50	79.92	6 for Dmax, 4 for D0.1cc
	47	–	64	–	Th2	3 × 20	IMRT	76%	68%	48.79	41.21	–	–	Intermediate
29	Male	58	C15.9	C79.5	C6-Th1	3 × 10	3‑D conformal	97%	97%	35.72	35.72	89.48	73.71	10.5 for Dmax, 3 for D0.1cc
	9	–	9	–	C6-Th1	3.5 × 16	IMRT	80%	64%	53.76	37.99	–	–	High (Dmax), low
30	Male	57	C64.9	C79.5	C2-Th2	3 × 10	3‑D conformal	106%	105%	41.18	40.56	103.3	88.67	13.5 for Dmax, 6 for D0.1cc
	41	–	70	–	C2	3.2 × 19	IMRT	86%	73%	62.12	48.11	–	–	High (Dmax), intermediate
31	Female	46	C20.9	C79.5	Th2‑9	2.5 × 15	3‑D conformal	104%	104%	44.85	44.85	92.96	86.71	7 for Dmax, 6 for D0.1cc
	9	–	6	–	Th3‑8	4 × 14	IMRT	71%	65%	48.11	41.86	–	–	High (Dmax), intermediate
32	Male	69	C22.1	C79.5	C2	2 × 20	3‑D conformal	102%	101%	41.21	40.60	89.01	83.46	6 for Dmax, 5 for D0.1cc
	26	–	7	–	C2	3.3 × 18	IMRT	73%	68%	47.80	42.86	–	–	Intermediate

*ReRT* re-irradiation, *SIB* simultaneous integrated boost, *BID* two fractions per day with 6 h interval, *EQD2* equivalent dose in 2‑Gy fractions, *IMRT* intensity-modulated radiotherapy, *Dmax* maximum dose

^a^SIB did not contribute, based on 2 Gy x 15

^b^only the first 23 fractions contributed

^c^boost contributed less (1.67 Gy per fraction)

^d^at start of re-irradiation

The most common fractionation regimen was 10 fractions of 3 Gy (21 of 64 treatment courses, 33%). Forty-eight of 64 treatment courses (75%) employed fraction sizes of 2.5 to 4 Gy to the target volume. In 22 cases (69%), the re-irradiation spinal cord equivalent Dmax was higher than that of the first treatment course. In most cases, Dmax was similar to D0.1cc (difference within 5%); however, in 15 of 64 treatment courses (23%) larger differences were registered, in line with the fact that many Japanese patients received cord-sparing intensity-modulated radiotherapy (IMRT), whereas the Norwegian center utilized simpler techniques with more homogeneous doses throughout the entire spinal canal.

The median cumulative spinal cord Dmax EQD2 was 80.7 Gy, minimum 61.12 Gy, maximum 114.79 Gy. The median cumulative spinal cord D0.1cc EQD2 was 76.1 Gy, minimum 61.12 Gy, maximum 95.62 Gy. Besides cumulative dose, other risk factors were present (single-course Dmax EQD2 ≥51 Gy in 9 patients, single-course D0.1cc EQD2 ≥51 Gy in 5 patients). The risk score (Table 1) was calculated both for Dmax and D0.1cc. For Dmax, 12 patients (37.5%) were low risk, 8 (25%) intermediate risk, and 12 (37.5%) high risk. For D0.1cc, 14 patients (44%) were low risk, 13 (41%) intermediate risk, and 5 (16%) high risk.

## Discussion

In contrast to previous publications that mainly included dosimetric data from the two-dimensional era without MRI-based spinal cord contouring [12, 13, 17], the present bi-institutional study attempted to assess the “true” spinal cord Dmax and D0.1cc. If the actual treatment planning did not include MRI and/or 3D dose–volume histograms, the respective plans were calculated by the authors in the context of this study. Ideally, the true dose–volume histograms would form the basis of future recommendations. Our group limited inclusion to patients re-irradiated to the cervical or thoracic spine. As already discussed by Sahgal et al. [18], the Dmax has a high degree of dose uncertainty, and therefore other dose–volume histogram parameters should also be analyzed. In a previous seminal paper, Sahgal et al. compared five cases of re-irradiation-induced myelopathy to a control group of 14 re-irradiated patients with 16 spinal segments treated [19]. In the small myelopathy cohort, the median EQD2 Dmax for the SBRT component and cumulative EQD2 were 61.7 Gy (range, 44.1–104.9 Gy) and 99.6 Gy (range, 77.2–154.9 Gy), respectively (α/β-value 2 Gy). In the cohort without myelopathy, the median EQD2 Dmax for the SBRT component and cumulative EQD2 were 12.5 Gy (range 1.9–58.7 Gy) and 52.4 Gy (range 39.1–111.2 Gy), respectively. For re-irradiation SBRT delivered in 1 to 5 fractions, Sahgal et al. have recommended that the cumulative thecal sac EQD2 Dmax should not exceed 70 Gy [19]. According to the older risk score, 75 Gy to the thecal sac would still result in a low risk of myelopathy, as long as the time interval is ≥6 months and neither of the two courses results in a single-course EQD2 ≥51 Gy [12, 13].

Both institutions reporting the present data chose to exceed previous recommendations in selected cases where the administration of lower doses to the target volume was not desirable (lack of local control) and where better cord sparing could not be achieved. Of course, such individual decisions require appropriate informed consent from the patients. Fortunately, radiation myelopathy has not been observed after re-irradiation courses that were considered intermediate or high risk according to the risk score displayed in Table 1 [12, 13]. For the low-risk group, the risk of myelopathy was indistinguishable from that of first-line radiotherapy in the previous reports (<5%) [12, 13] and zero in the present study, which thereby validates the original findings. If one puts aside the methodological differences between the previous and the present reports, and chooses to add the new intermediate-risk patients to the 8 previous patients (2/8 had radiation myelopathy for a risk of 25%), the new risk estimate would read 2/16 (based on Dmax) and 2/21 (based on D0.1cc), respectively. The resulting risks of 12.5 and 9.5%, respectively, would then appear lower than previously estimated. We feel that the new results lend support to the authors’ current clinical practice of loosening the dose constraints for spinal cord re-irradiation if lower doses cannot be achieved, despite the limited number of patients eligible for this study. Importantly, all excluded patients who died within 6 months from re-irradiation or have shorter, ongoing follow-up also remained free from myelopathy. This finding strengthens our current policy and leads us to believe that we are not gambling with patient safety. The pros and cons of a conservative, low-myelopathy-risk dose prescription and a possibly slightly more risky “prioritize local control” prescription that involves higher doses must be explained to the patients in sufficient detail. In the Japanese re-irradiation study, which also included patients with short follow-up and/or lumbosacral re-irradiation, the 3‑year local control rate was 84% [14]. This figure is in line with other results in the literature [4].

In clinical routine, different treatment planning and delivery techniques should be considered when preparing a new patient for treatment to make sure one avoids unnecessary risks, e.g., by achieving steep dose gradients so that only a small volume of the spinal cord receives a high cumulative dose. It is also necessary to minimize the risk of geographical miss, ensuring that high-dose areas do not move in unintended ways [20]. Despite several advantages of the present study compared to its predecessors, limitations must also be considered. We acknowledge that several of these are present, including the retrospective design, the limited number of eligible participants, their heterogeneous baseline and treatment characteristics, and the uncertainty of reconstructed dose distributions. Most re-irradiation courses employed moderate hypofractionation rather than typical SBRT fractionation. Of course, patients whose follow-up is still ongoing may be at risk of radiation myelopathy at later timepoints. Despite several experimental approaches, this severe complication, which may still be observed in the clinic [21], is difficult to modulate pharmacologically [22–24]. Hopefully, our study will encourage other institutions to publish their experiences with spinal re-irradiation, because further research is needed to confirm the limited tolerance data.

## Conclusion

Even patients treated to higher cumulative doses than previously recommended, or at considerable risk of myelopathy according to a published risk score, remained free from this complication, although one must acknowledge the potential for subsequent manifestation of damage in patients currently alive, i.e., still at risk while being followed. Individualized decisions to re-irradiate after appropriate informed consent are an acceptable strategy, including scenarios where low re-irradiation doses to the spinal cord would compromise target coverage and tumor control probability to an unacceptable degree.
